# Supplementary material for: GPR56/ADGRG1 is associated with response to antidepressant treatment
Source: Nat Commun. 2020 Apr 2;11:1635. doi: 10.1038/s41467-020-15423-5 (PMC7118175; doi:10.1038/s41467-020-15423-5)
Supplement: Supplementary file 1 — Supplementary Information [file 41467_2020_15423_MOESM1_ESM.pdf]

GPR56/ADGRG1 is associated with response to antidepressant treatment

Belzeaux et al.

Supplementary Information

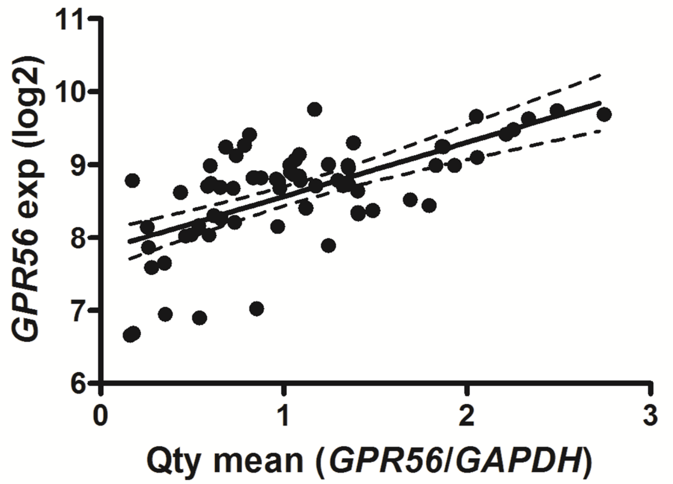

Supplementary Figure 1: Technical validation of *GPR56* expression in the discovery cohort. We technically validated our microarray data in a representative sub-group of samples (n=69) using qRT-PCR. Our results revealed a significant correlation across quantification methods (two-sided Pearson correlation=0.64, p=4.05E-9). Source data are provided as a Source Data file.

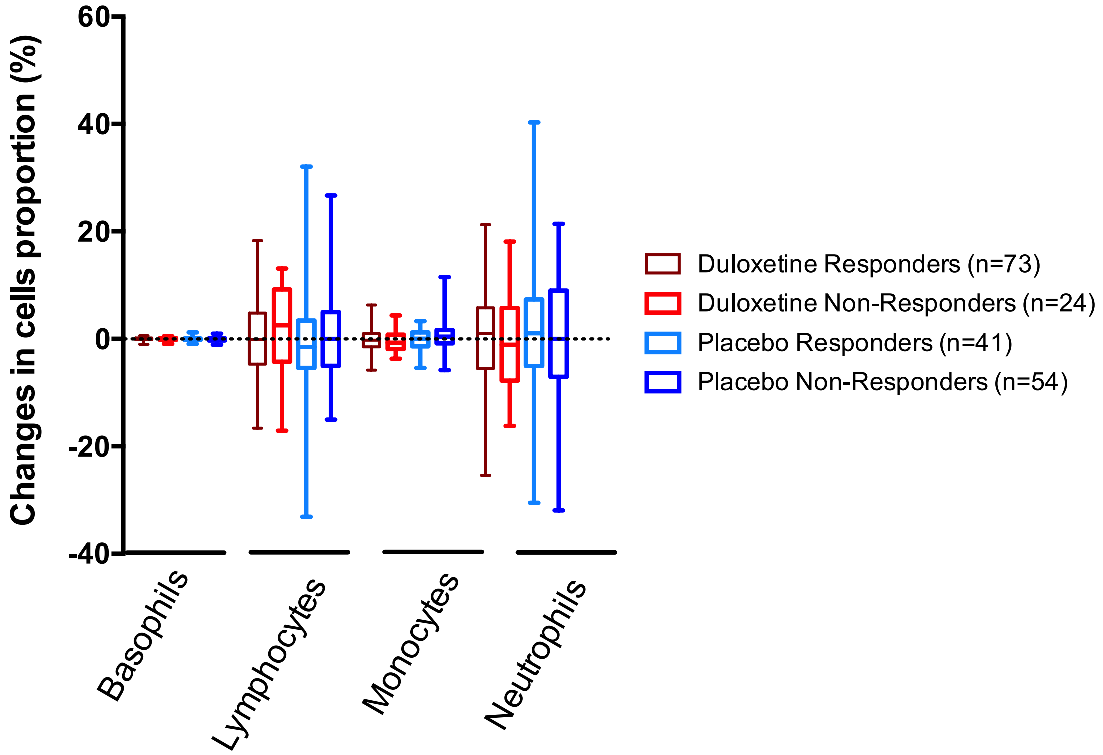

Supplementary Figure 2: Assessment of potential biases related to differential cellular composition of group samples. In the discovery cohort, we analyzed cell counts from haematological data including the proportion of monocytes, lymphocytes and neutrophils in the total leukocytic population. We observed no significant differences in cell proportions between placebo and duloxetine groups at time of inclusion, or variation of cell proportions during treatment according to treatment or according to response. Moreover, using GLM for repeated measures, we found no significant time x group x response interaction for each of the cell types. Graph represents Box and Whiskers Min to Max. Source data are provided as a Source Data file.

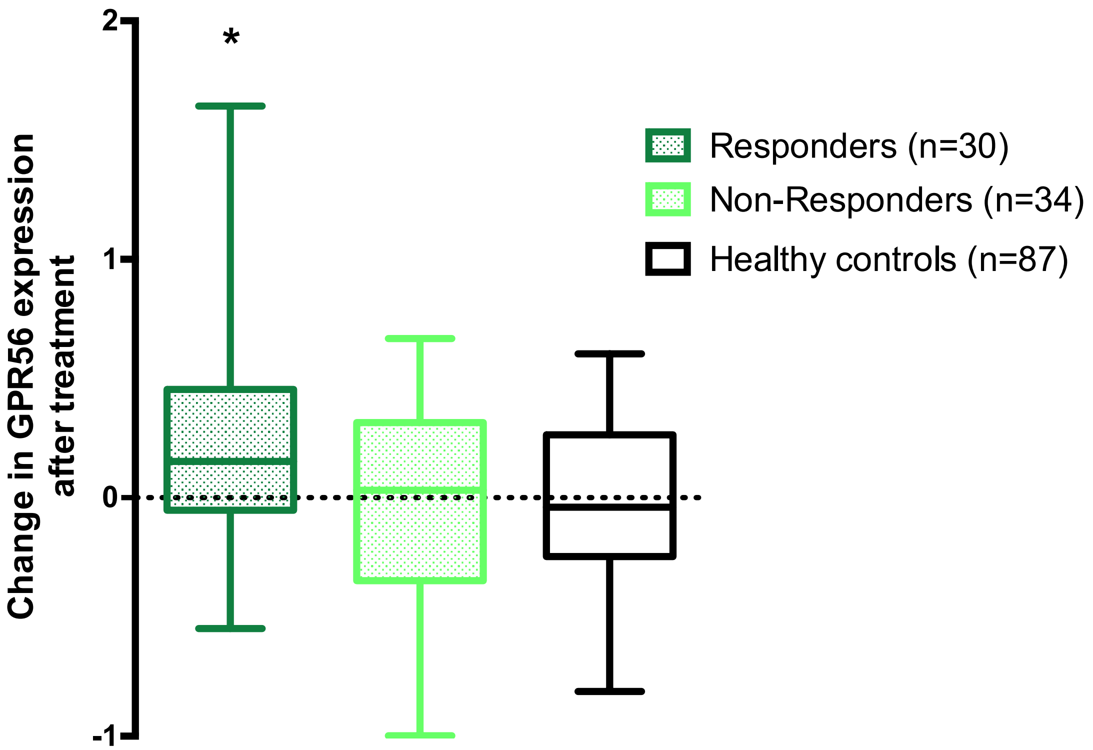

Supplementary Figure 3: Replication of *GPR56* mRNA increase after response in Marseille cohort. *GPR56* mRNA was increased only in responder patients in comparison to healthy controls and non-responder patients at 8 weeks (GLM for repeated measure including Week 0 and Week 8 evaluation, $F(2,148)=4.98$, $p=0.008$). Graph represents Box and Whiskers Min to Max. Source data are provided as a Source Data file.

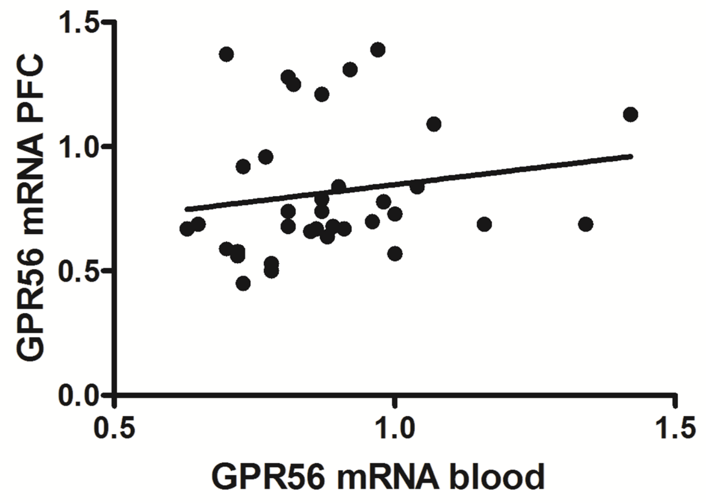

Supplementary Figure 4: Prefrontal cortex (PFC) and peripheral *GPR56* mRNA levels were significantly correlated in stressed mice. Pearson correlations (two-sided) were performed between *GPR56* expression in PFC and whole blood (n=23; r=0.51; p=0.018). Source data are provided as a Source Data file.

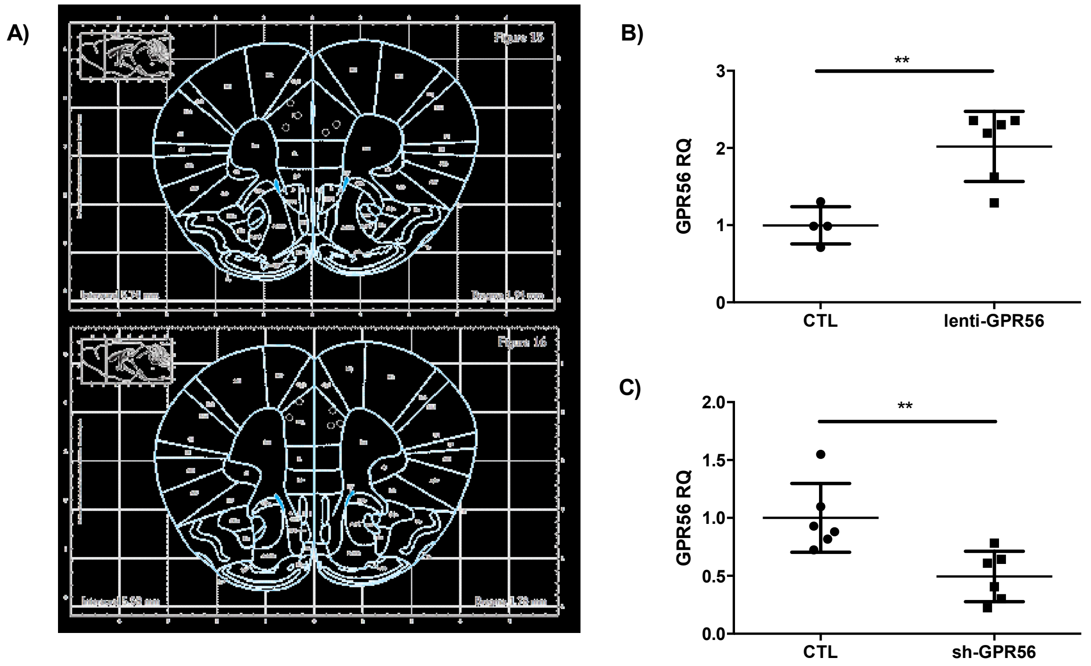

Supplementary Figure 5: Lentiviral injections into mouse prefrontal cortex.

(A) Hit maps of lentiviral injections into mouse PFC. At the end of the behavioral experiments, a subgroup of mice was used to assess histologically the site of the injection. Images depict representative injection sites according to the atlas of [1].

(B) Expression of *Gpr56* in injected mice. In naïve mice, bilateral PFC infusions of a lentivirus-*Gpr56* construct (n=6 mice) resulted in PFC *Gpr56* upregulation in comparison to controls (n=4 mice). *Gpr56* expression was assessed by quantitative real time PCR (qRT-PCR) using *26S* as an endogenous control, and was significantly upregulated in lenti-*Gpr56* injected mice (FC=2.02, two-sided t=4.09, p=0.003).

(C) Expression of *Gpr56* in injected mice. In naïve mice, bilateral PFC infusions of a lentivirus-sh-*Gpr56* construct (n=6 mice) resulted in PFC *Gpr56* downregulation in comparison to controls (n=6 mice). *Gpr56* expression was assessed by quantitative real time PCR (qRT-PCR) using *26S* as an endogenous control, and was significantly downregulated in sh-*Gpr56* injected mice (FC=0.49, two-sided t=3.37, p=0.007).

Error bars represent standard deviation of the mean. Source data are provided as a Source Data file.

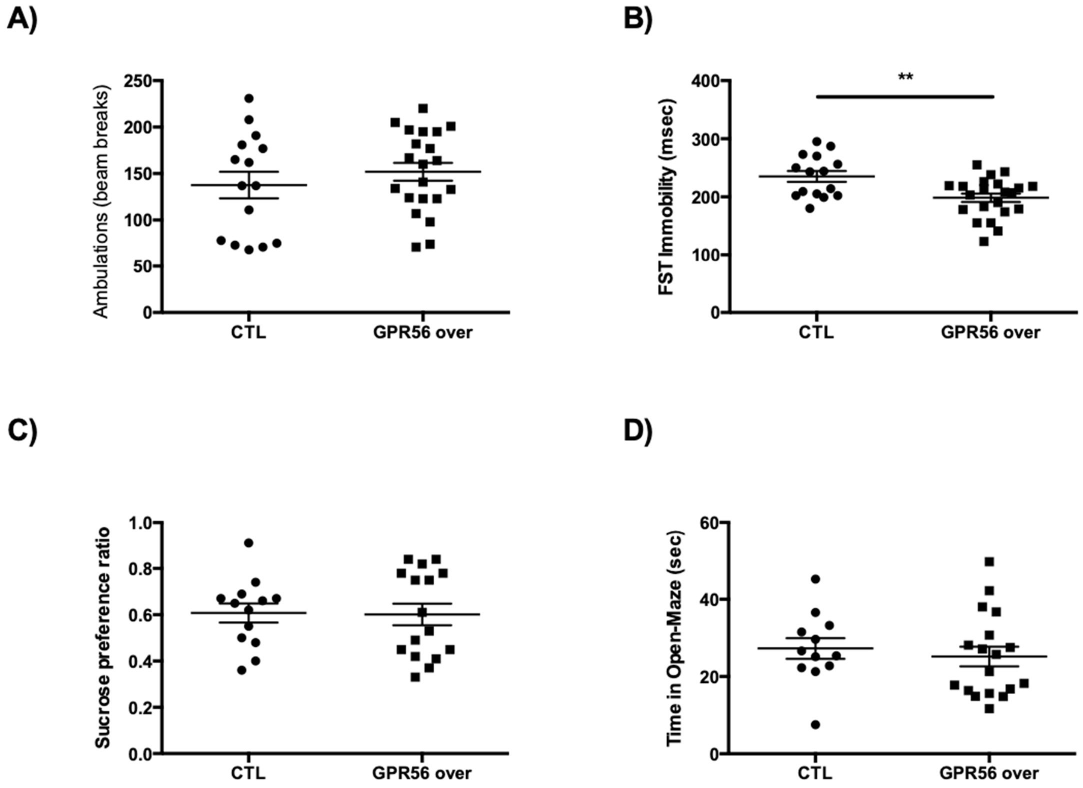

Supplementary Figure 6: The behavioral effects of *Gpr56* overexpression in mice. Mice injected in the PFC with a *Gpr56*-expressing lentivirus (GPR56-over) or control virus (CTL), underwent behavioral tests after 4-5 weeks to assess (A) locomotion (GPR56-over n=21 mice and CTL n=15 mice), (B) depressive-like behavior (forced swimming test, FST) (GPR56-over n=22 mice and CTL n=15 mice, two-sided t=3.115, p=0.0033, (C) anhedonia (sucrose preference test) (GPR56-over n=16 mice and CTL n=13 mice), and (D) anxiety (O-maze) (GPR56-over n=18 mice and CTL n=12 mice). Error bars represent standard deviation of the mean. Source data are provided as a Source Data file.

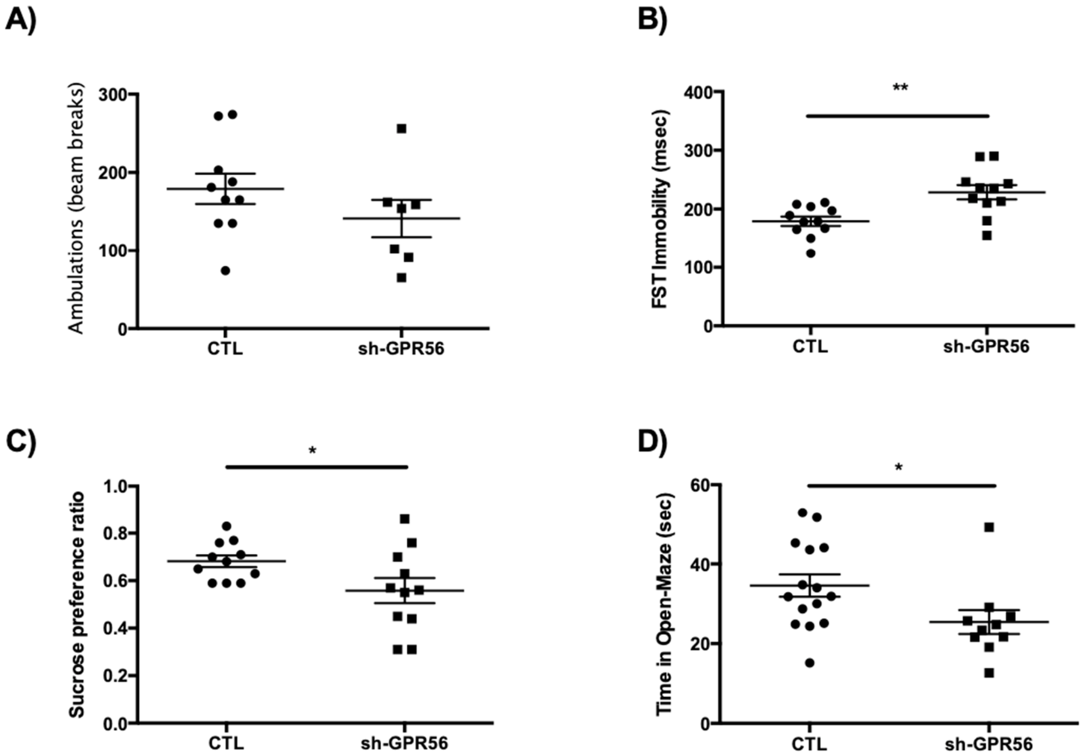

Supplementary Figure 7: The behavioral effects of *Gpr56* knockdown in mice. Mice injected in the PFC with a sh-*Gpr56*-expressing lentivirus (sh-GPR56) or control virus (CTL), underwent behavioral tests after 4-5 weeks to assess (A) locomotion (sh-GPR56 n=7 mice and CTL n=10 mice), (B) depressive-like behavior (forced swimming test, FST) (sh-GPR56 n=11 mice and CTL n=11 mice) two-sided t=3.368, p=0.0031, (C) anhedonia (sucrose preference test) (sh-GPR56 n=11 mice and CTL n=11 mice) two-sided t=2.126, p=0.046, and (D) anxiety (O-maze) (sh-GPR56 n=10 mice and CTL n=15 mice) two-sided t=2.163, p=0.041. Error bars represent standard deviation of the mean. Source data are provided as a Source Data file.

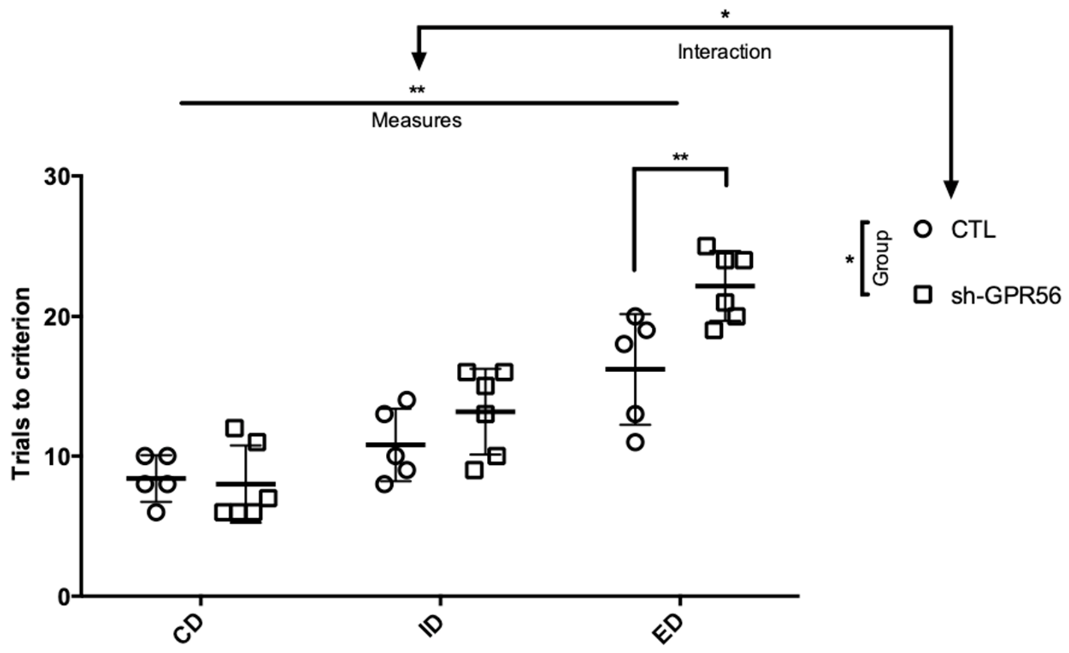

Supplementary Figure 8: Effects of *Gpr56* knockdown on cognitive function. Mice injected in the PFC with a sh-*Gpr56*-expressing lentivirus (sh-GPR56, n=6) or control virus (CTL, n=5), underwent behavioral tests after 4-5 weeks to asses cognitive functioning. Attentional set shifting test demonstrated alterations in cognitive function related to PFC functioning in sh-*Gpr56* mice. Repeated measures ANOVA and post-hoc test demonstrated an effect of group (sh-*Gpr56* vs CTL, F(2,18)=6.64, p=0.030), an effect of cognitive task (p<0.001) and an interaction (p=0.048), with significant differences only for extra-dimensional shift between groups (two-sided p=0.009). Error bars represent standard error of the mean. Source data are provided as a Source Data file.

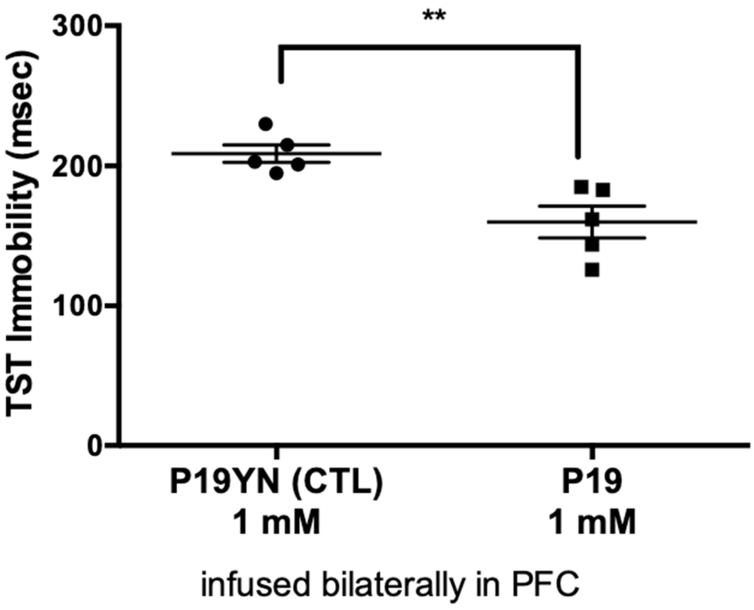

Supplementary Figure 9: GPR56 agonist P19 peptide infused bilaterally in the PFC (1 mM) (n=5 mice) decreased immobility time and demonstrated antidepressant-like effects in comparison to controls (CTL, n=5 mice) (two-sided t=3.775, p=0.006)**.** Source data are provided as a Source Data file.

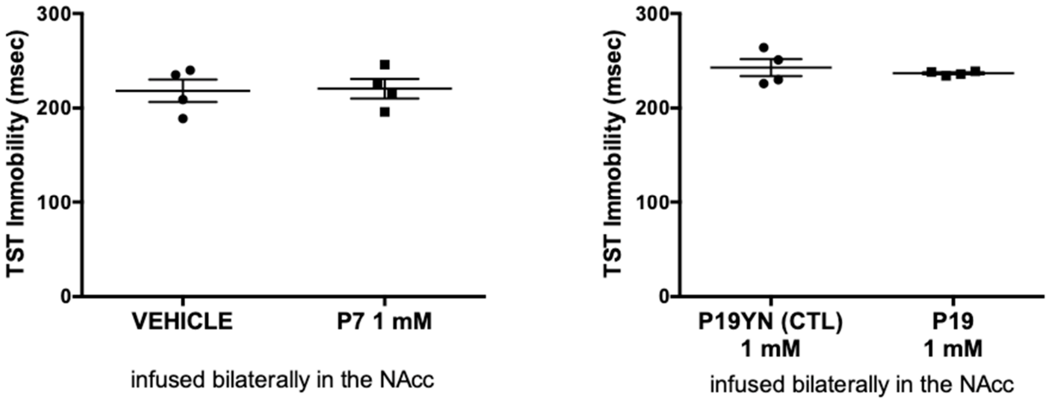

Supplementary Figure 10: Effect of infusion of peptide agonists P7 (n=4 mice) or vehicle (n=4 mice), and P19 (n=4 mice) or P19YN (n=4 mice) into the nucleus accumbens (NAcc) on depressive-like behavior. Source data are provided as a Source Data file.

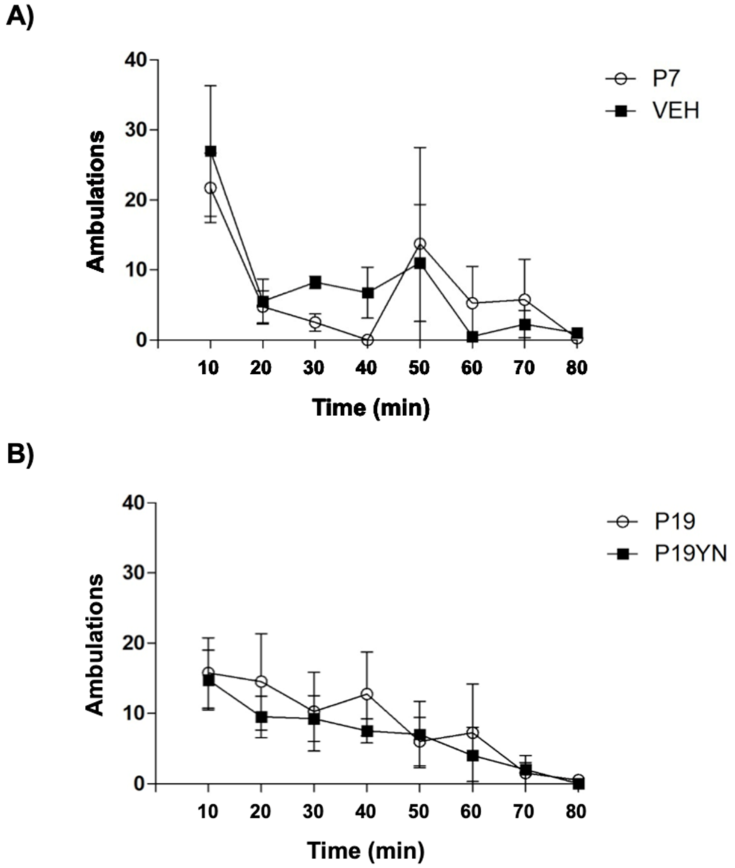

Supplementary Figure 11: Effect of peptide agonists infused into the PFC on basic locomotion. (**A**) n=4 mice injected with P7 in comparison to vehicle (VEH, n=4 mice) and (**B**) n=4 mice injected with P19 in comparison to P19YN (n=4 mice). Error bars represent standard error of the mean. Source data are provided as a Source Data file.

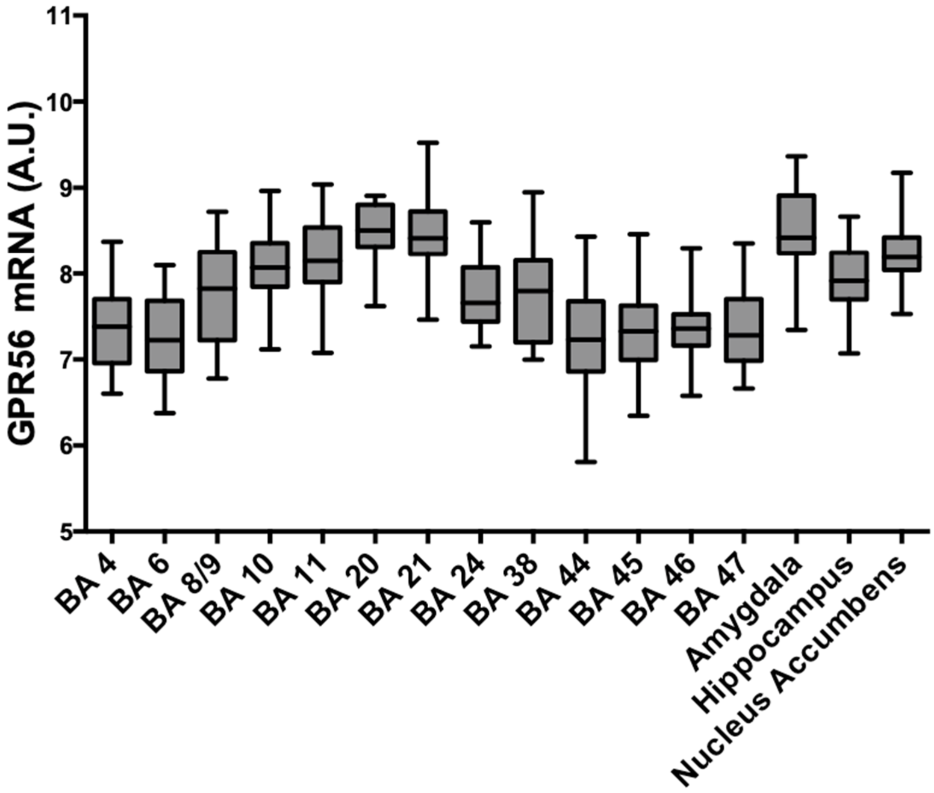

Supplementary Figure 12: Expression of *GPR56* across human brain regions based on microarray analysis in Brodmann area (BA) 4 (n=20 samples), BA 6 (n=26 samples), BA 8/9 (n=19 samples), BA 10 (n=19 samples), BA 11 (n=20 samples), BA 20 (n=16 samples), BA 21 (n=19 samples), BA 24 (n=20 samples), BA 38 (n=18 samples), BA 44 (n=30 samples), BA 45 (n=28 samples), BA 46 (n=24 samples), BA 47 (n=28 samples), Amygdala (n=27 samples), Hippocampus (n=21 samples) and Nucleus Accumbens (n=21 samples) demonstrated a homogeneous level of expression across brain regions. (Coefficient of Variation=0.08). Source data are provided as a Source Data file.

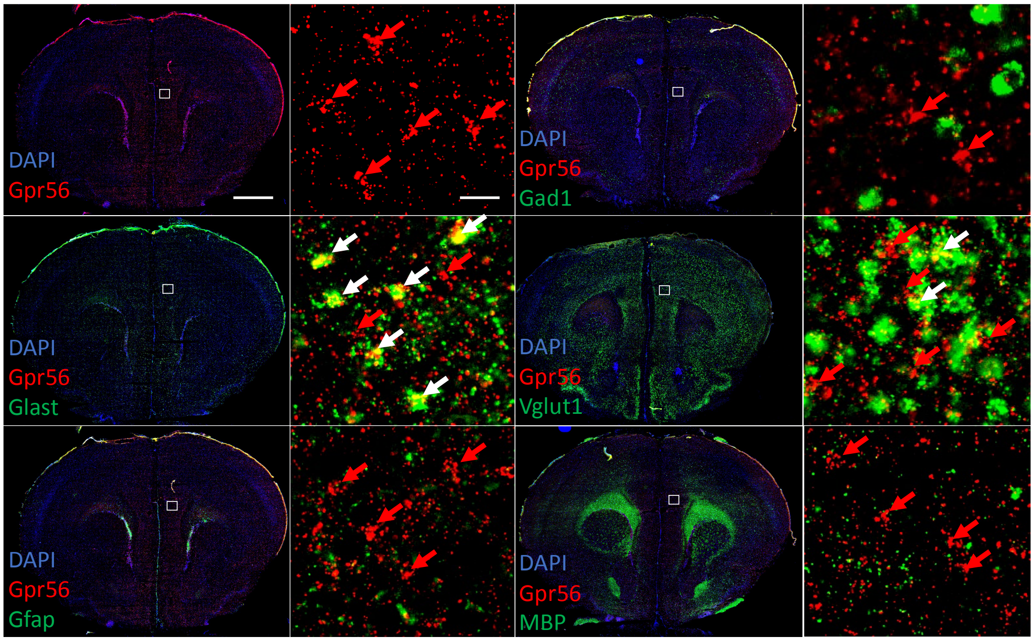

Supplementary Figure 13: Fluorescence *in situ* hybridization to determine cell-specific expression of Gpr56 in mouse PFC. GPR56 was co-localized with Glast (astrocyte-specific marker, white arrow in the panel) and Vglut1 (glutamatergic neuron-specific marker, white arrow in the panel). White scale bars represent 1mm for whole slices (depicted upper left) and 25µm for magnification panels (depicted upper right). Experiment was repeated independently three times with similar results.

	Duloxetine Arm (N=112)		Placebo Arm (N=125)				P-value[1]
	Mean	SD	Mean	SD	t	df	
**Age (Years)**	47.5	12.9	46.1	12.8	-0.739	235	0.431
**Body Mass Index (kg/m^2)**	25.7	5.2	25.6	4.9	0.116	235	0.908
**MADRS[2] at baseline**	31.0	3.5	31.4	3.9	0.697	235	0.486
**MADRS[2] after treatment**	10.4	7.8	18.4	10.5	6.785	227.7	<0.001
	N	%	N	%	Value	df	
**Female gender**	81	72.3%	84	67.2%	0.733	1	0.392
**First MDE**	7	6.3%	12	9.6%	0.899	1	0.343
**Response after treatment**	89	79.5%	51	40.8%	36.53	1	<0.001

[1] Two-sided t-test for continuous variables and Chi-2 test for categorical variables
[2] Montgomery Åsberg Depression Rating Scale

Supplementary Table 1: Patient characteristics in discovery cohort. SD: standard deviation, df: degrees of freedom

Gene Name		PROBE_ID	FC	duloxetine R	duloxetine NR	placebo R	placebo NR
**GPR56**	G protein-coupled receptor 56	ILMN_2352097	1.19	4.21E-04	p>0.05	p>0.05	p>0.05
**TAF15**	TAF15 RNA polymerase II, TATA box binding protein (TBP)-associated factor, 68kDa	ILMN_1678707	1.18	2.85E-05	p>0.05	p>0.05	p>0.05
**GPR56**	G protein-coupled receptor 56	ILMN_2384122	1.17	1.98E-03	p>0.05	p>0.05	p>0.05
**PTGDS**	prostaglandin D2 synthase 21kDa (brain)	ILMN_1664464	1.14	4.38E-04	p>0.05	p>0.05	p>0.05
**LOC729978**	similar to LOC339047 protein	ILMN_3304111	1.14	4.07E-04	p>0.05	p>0.05	p>0.05
**LOC23117**	KIAA0220-like protein	ILMN_1656868	1.13	5.76E-04	p>0.05	p>0.05	p>0.05
**CECR1**	cat eye syndrome chromosome region, candidate 1	ILMN_1751851	1.13	1.81E-04	p>0.05	p>0.05	p>0.05
**RASAL3**	RAS protein activator like 3	ILMN_1795089	1.13	1.40E-04	p>0.05	p>0.05	p>0.05
**TBC1D9B**	TBC1 domain family, member 9B (with GRAM domain)	ILMN_2850537	1.12	8.68E-03	p>0.05	p>0.05	p>0.05
**SMARCC2**	SWI/SNF related, matrix associated, actin dependent regulator of chromatin, subfamily c, member 2	ILMN_1773620	1.12	1.17E-04	p>0.05	p>0.05	p>0.05
**FTSJ3**	FtsJ homolog 3 (E, coli)	ILMN_1811692	1.12	9.87E-05	p>0.05	p>0.05	p>0.05
**CSTF3**	cleavage stimulation factor, 3' pre-RNA, subunit 3, 77kDa	ILMN_1762002	1.12	1.82E-04	p>0.05	p>0.05	p>0.05
**AOF2**	amine oxidase (flavin containing) domain 2	ILMN_1813840	1.12	1.23E-04	p>0.05	p>0.05	p>0.05
**MORC2**	MORC family CW-type zinc finger 2	ILMN_2103591	1.11	3.27E-04	p>0.05	p>0.05	p>0.05
**NCL**	nucleolin	ILMN_2121437	1.11	2.06E-05	p>0.05	p>0.05	p>0.05
**SBF1**	SET binding factor 1, transcript variant 1	ILMN_1703246	1.11	8.45E-05	p>0.05	p>0.05	p>0.05
**EP400**	E1A binding protein p400	ILMN_1673023	1.11	4.71E-05	p>0.05	p>0.05	p=0.036, increased
**YEATS2**	YEATS domain containing 2	ILMN_1676899	1.11	1.18E-05	p>0.05	p>0.05	p>0.05
**INO80**	INO80 homolog (S. cerevisiae)	ILMN_1678362	1.11	4.53E-04	p>0.05	p>0.05	p>0.05
**PLCG1**	phospholipase C, gamma 1	ILMN_2382906	1.11	2.32E-03	p>0.05	p>0.05	p>0.05
**SRRM2**	serine/arginine repetitive matrix 2	ILMN_1734602	1.11	1.16E-04	p>0.05	p>0.05	p>0.05
**HCFC1**	host cell factor C1 (VP16-accessory protein)	ILMN_1732705	1.11	3.08E-04	p>0.05	p>0.05	p>0.05
**FAM125B**	family with sequence similarity 125, member B	ILMN_1652525	1.11	8.28E-05	p>0.05	p>0.05	p>0.05
**CLEC16A**	C-type lectin domain family 16, member A	ILMN_1781752	1.10	1.35E-04	p>0.05	p>0.05	p=0.047, increased

**DNMT1**	DNA (cytosine-5-)-methyltransferase 1	ILMN_1760201	1.10	2.96E-05	p>0.05	p>0.05	p>0.05
**PPRC1**	peroxisome proliferator-activated receptor gamma, coactivator-related 1	ILMN_1796210	1.10	5.94E-04	p>0.05	p>0.05	p>0.05
**SFRS14**	splicing factor, arginine/serine-rich 14	ILMN_1711270	1.10	3.05E-05	p>0.05	p>0.05	p>0.05
**PHRF1**	PHD and ring finger domains 1	ILMN_3245476	1.10	1.51E-04	p>0.05	p>0.05	p>0.05
**IPO9**	importin 9	ILMN_1723117	1.10	1.96E-04	p>0.05	p>0.05	p>0.05
**TAF1C**	TATA box binding protein (TBP)-associated factor, RNA polymerase I, C, 110kDa	ILMN_2940707	1.10	2.70E-04	p>0.05	p>0.05	p>0.05
**ZC3H5**	zinc finger CCCH-type containing 5	ILMN_1689119	1.10	1.31E-04	p>0.05	p>0.05	p>0.05
**FLAD1**	FAD1 flavin adenine dinucleotide synthetase homolog (S. cerevisiae)	ILMN_1663667	1.10	1.04E-04	p>0.05	p>0.05	p>0.05
**UCKL1**	uridine-cytidine kinase 1-like 1	ILMN_2050255	1.10	9.41E-06	p>0.05	p>0.05	p>0.05
**FLJ10081**	hypothetical protein FLJ10081	ILMN_1745217	1.10	5.01E-05	p>0.05	p>0.05	p>0.05
**HNRPM**	heterogeneous nuclear ribonucleoprotein M	ILMN_1745385	1.10	9.69E-06	p>0.05	p>0.05	p>0.05
**BMS1**	BMS1 homolog, ribosome assembly protein (yeast)	ILMN_1772713	1.09	1.24E-04	p>0.05	p>0.05	p>0.05
**TRRAP**	transformation/transcription domain-associated protein	ILMN_1660368	1.09	2.51E-05	p>0.05	p>0.05	p>0.05
**LOC100130919**	hypothetical protein LOC100130919	ILMN_3176090	1.09	6.96E-06	p>0.05	p>0.05	p>0.05
**FRAP1**	FK506 binding protein 12-rapamycin associated protein 1	ILMN_1769031	1.09	1.43E-05	p>0.05	p>0.05	p>0.05
**LOC642073**	similar to MHC class II antigen	ILMN_3243714	1.08	2.02E-04	p>0.05	p>0.05	p>0.05
**HLA-DRB5**	major histocompatibility complex, class II, DR beta 5	ILMN_1697499	1.06	4.02E-03	p>0.05	p>0.05	p>0.05
**HLA-DRB1**	major histocompatibility complex, class II, DR beta 1	ILMN_1715169	1.05	8.02E-04	p>0.05	p>0.05	p>0.05

Supplementary Table 2: Gene expression differences between responders and non-responders in the discovery cohort. Fold change (FC) is for T8/T0 for duloxetine responders. P-values represent differential expression between T0 and T8 (two-sided, no adjustment for multiple comparisons). NR: non-responders, R: responders.

Specific treatments	N	%
SSRI	31	48.4
SNRI	23	35.9
Tricyclic	6	9.4
MAOI	1	1.6
Other antidepressant	7	10.9
ECT	1	1.6
Atypical antipsychotic	16	25
Typical antipsychotic	10	15.6
Lithium	5	7.8
Valproate	1	1.6
Carbamazepine	2	3.1
**Number of medications (with the exception of anxiolytic/hypnotic)**		
1 medication	38	59.4
2 medications	20	31.3
more than 2	6	9.3
**Anxiolytic / Hypnotic**	57	89.1

Supplementary **Table 3: Treatments received in the Marseille cohort.**

	SOURCE	PATHWAY SIZE	Enrichment Score	Normalized Enrichment Score	p-val	FWER p-val
Gene Set up-regulated by GPR56 AGONIST						
AKT PATHWAY	BIOCARTA	19	-0.60	-2.38	<0.001	0.02
EIF4 PATHWAY	BIOCARTA	23	-0.57	-2.14	<0.001	0.08
GSK3 PATHWAY	BIOCARTA	25	-0.60	-2.12	0.01	0.09
VIRAL GENOME REPLICATION	GO Biological Process GO:0019079	23	-0.47	-2.10	<0.001	0.11
TFF PATHWAY	BIOCARTA	19	-0.57	-2.06	0.00	0.15
Gene Set down-regulated by GPR56 AGONIST						
POLY(A)+ MRNA EXPORT FROM NUCLEUS	GO Biological Process GO:0016973	15	0.72	2.43	<0.001	0.01
ESTABLISHMENT OF SPINDLE LOCALIZATION	GO Biological Process GO:0051293	44	0.49	2.12	0.00	0.10
PROTEIN K48-LINKED UBIQUITINATION	GO Biological Process GO:0070936	52	0.43	2.09	0.02	0.12
POSITIVE REGULATION OF LAMELLIPODIUM ORGANIZATION	GO Biological Process GO:1902745	26	0.61	2.05	<0.001	0.17

Supplementary Table 4: Gene Set Enrichment Analysis described several gene sets associated with the transcriptional signature of GPR56 agonists in neuroblastoma cells (FWER p-value < 0.20). p-values were calculated by using an empirical phenotype-based permutation test procedure from GSEA [2] and were adjusted for multiple testing using Family-Wise Error Rate (FWER).

Supplementary References

1. Paxinos G, Franklin KBJ. *The mouse brain in stereotaxic coordinates*, 2nd edn. Academic Press (2001).

2. Benjamini Y, Hochberg Y. Controlling the False Discovery Rate: A Practical and Powerful Approach to Multiple Testing. *Journal of the Royal Statistical Society Series B (Methodological)* **57**, 289-300 (1995).
