# Supplementary material for: GPR56/ADGRG1 is associated with response to antidepressant treatment
Source: Nat Commun. 2020 Apr 2;11:1635. doi: 10.1038/s41467-020-15423-5 (PMC7118175; doi:10.1038/s41467-020-15423-5)
Supplement: Supplementary file 3 — Reporting summary [file 41467_2020_15423_MOESM3_ESM.pdf]

nature research

Corresponding author(s): Gustavo TURECKI

Last updated by author(s): Feb 11, 2020

Reporting Summary

Nature Research wishes to improve the reproducibility of the work that we publish. This form provides structure for consistency and transparency in reporting. For further information on Nature Research policies, see Authors & Referees and the Editorial Policy Checklist .

Statistics

For all statistical analyses, confirm that the following items are present in the figure legend, table legend, main text, or Methods section.

n/a	Confirmed	
☐	☒	The exact sample size (*n*) for each experimental group/condition, given as a discrete number and unit of measurement
☐	☒	A statement on whether measurements were taken from distinct samples or whether the same sample was measured repeatedly
☐	☒	The statistical test(s) used AND whether they are one- or two-sided<br>*Only common tests should be described solely by name; describe more complex techniques in the Methods section.*
☐	☒	A description of all covariates tested
☐	☒	A description of any assumptions or corrections, such as tests of normality and adjustment for multiple comparisons
☐	☒	A full description of the statistical parameters including central tendency (e.g. means) or other basic estimates (e.g. regression coefficient) AND variation (e.g. standard deviation) or associated estimates of uncertainty (e.g. confidence intervals)
☐	☒	For null hypothesis testing, the test statistic (e.g. *F*, *t*, *r*) with confidence intervals, effect sizes, degrees of freedom and *P* value noted<br>*Give P values as exact values whenever suitable.*
☒	☐	For Bayesian analysis, information on the choice of priors and Markov chain Monte Carlo settings
☒	☐	For hierarchical and complex designs, identification of the appropriate level for tests and full reporting of outcomes
☐	☒	Estimates of effect sizes (e.g. Cohen's *d*, Pearson's *r*), indicating how they were calculated

Our web collection on statistics for biologists contains articles on many of the points above.

Software and code

Policy information about availability of computer code

Data collection	Excel 2013, Epidata V3.1
Data analysis	Flexarray V1.6.3, MultiExperiment Viewer 4 (TM4 software suite), SPSS V21, GraphPad Prism 5, QuantStudioTM Real-Time PCR Software v1.1, FASTX toolkit (v0.0.14), trimmomatic (v0.36), TopHat (v2.1.1) (using Bowtie2 (v2.2.9))

For manuscripts utilizing custom algorithms or software that are central to the research but not yet described in published literature, software must be made available to editors/reviewers. We strongly encourage code deposition in a community repository (e.g. GitHub). See the Nature Research guidelines for submitting code & software for further information.

Data

Policy information about availability of data

All manuscripts must include a data availability statement. This statement should provide the following information, where applicable:

- Accession codes, unique identifiers, or web links for publicly available datasets
- A list of figures that have associated raw data
- A description of any restrictions on data availability

The source data (raw data) underlying figures 1A-C, 2B-C, 3B-E, supplementary figures 1 to 12 and supplementary table 1 and 2 are provided as a source data file.
We used reference genome in RNA-seq analysis: GRCh38 , https://www.ncbi.nlm.nih.gov/assembly/GCF_000001405.39

Field-specific reporting

Please select the one below that is the best fit for your research. If you are not sure, read the appropriate sections before making your selection.

Life sciences study design

All studies must disclose on these points even when the disclosure is negative.

Sample size

For the discovery cohort, there was no prior statistical determination of sample size. However, using the ssize.fdr R/CRAN package with the following parameters: an estimated standard deviation (% = 0.8), a minimum effect size (! = 1.2), a false discovery rate, FDR (" = 0.05), a desired power (1-# = 0.9), a minimum number of 20 samples by group is needed for $0 (the proportion of transcripts that are not differentially expressed) = 0.95. Therefore, based on this calculation and previous publications focusing on gene expression variation associated with antidepressant response, we considered that the discovery sample size is sufficient.
For the post-mortem brain study, we determined sample size taking in consideration previous publications and sample availability in particular based on Lopez JP, et al. Nat Med 2014 and Nat Med and Apazoglou K, et al. Nat Med 2018. In these papers, sample size was < 20 subjects per group, as a consequence we considered that sample size > 20 per group should be sufficient.
For animal studies, there was no prior statistical determination of sample size but sample sizes are consistent to those reported in the literature in similar studies (Lopez JP, et al. Nat Med 2014 and Nat Med and Apazoglou K, et al. Nat Med 2018).

Data exclusions

For micro-array analysis in discovery cohort, a principal component analysis was used to identify outliers resulting in identification and exclusion of 13 samples. No samples or data were excluded from the replication cohorts.
For animal studies: (i) In chronic animal study protocols (UCMS, phenotyping after viral injections) mice were excluded from the statistical analysis if they died or became seriously ill during the protocol ; (ii) in all behavioral experiments mice were excluded from the analysis if their behavior was incompatible with the aim of the test (i.e. mice climbing over their tail in the TST; mice that did not drink at all during the sucrose preference test). These criteria were preestablished based on previous experience and pilot experiments.

Replication

The clinical study includes two distinct replication cohorts involving different sites/countries.
Animal studies: Behavioral studies discriminating responder versus nonresponder mice in a protocol involving UCMS followed by antidepressant (fluoxetine) administration have been replicated in three cohorts. All other determinations involved one cohort.
All PCR experiments were conducted in triplicate except for the Marseille replication cohort (duplicate).
All attempts at replication were successful.

Randomization

Patients from the discovery cohort were randomized to double-blind treatment with either duloxetine (60mg die, N=112), a serotonin norepinephrine reuptake inhibitor (SNRI), or placebo (N=125).
First replication cohort (Montréal cohort) was an open label trial. All patients were treated with citalopram and no randomization was possible. Group allocation was made prospectively at the end of the follow-up (responders or non responders).
The third cohort (Marseille cohort) was naturalistic. Group allocation was made prospectively at the end of the follow-up.
For post-mortem brain samples, group allocation was made after a psychological autopsy. Age, sex, post-mortem interval, tissue pH were used as co-variates.
No randomization table was used in animal studies. All animals were male adults (3-6 months of age) that were commercially purchased and assigned pseudorandomly to experimental groups upon arrival.

Blinding

The discovery cohort was double-blind. All depressed participants in both replication cohorts were treated with antidepressants (open label).
All gene expression measurements were conducted by a researcher blinded to group allocation.
Behavioral tests were run by a blinded observer. Behavioral scoring was done by a second blinded observer.

Reporting for specific materials, systems and methods

We require information from authors about some types of materials, experimental systems and methods used in many studies. Here, indicate whether each material, system or method listed is relevant to your study. If you are not sure if a list item applies to your research, read the appropriate section before selecting a response.

Materials & experimental systems

n/a	Involved in the study	
x		Antibodies
	x	Eukaryotic cell lines
x		Palaeontology
	x	Animals and other organisms
	x	Human research participants
x		Clinical data

Methods

n/a	Involved in the study	
x		ChIP-seq
x		Flow cytometry
x		MRI-based neuroimaging

Eukaryotic cell lines

Policy information about cell lines

Cell line source(s)	Human neuroblastoma cells (SK-N-AS) were obtained from ATCC (CRL-2137)
Authentication	Cells were authenticated by the manufacturer by morphology, chromosome analysis and/or differentiation potential.
Mycoplasma contamination	Cell lines were confirmed by the manufacturer's to be free from mycoplasma contamination by Hoecht DNA stain and Agar culture.
Commonly misidentified lines (See ICLAC register)	None

Animals and other organisms

Policy information about studies involving animals; ARRIVE guidelines recommended for reporting animal research

Laboratory animals	Mice, C57Bl6 and BALB/cJico male adults (3-6 months old). The mice were kept under standard conditions at 22±1°C, and a 12 hour light-dark cycle with food and water available ad libitum except when food/water deprivation was part of the experimental protocol. Humidity levels were between 45% and 55%.
Wild animals	n/a
Field-collected samples	n/a
Ethics oversight	All animal protocols and welfare complied with French and European Ethical regulations. The experimental protocols were approved by the local Ethical Committee (Comité d'éthique en expérimentation animale Charles Darwin N5).

Note that full information on the approval of the study protocol must also be provided in the manuscript.

Human research participants

Policy information about studies involving human research participants

Population characteristics	The discovery cohort consisted of 237 patients in a MDE (69.6 % female) who were randomized to double-blind treatment with either duloxetine (60mg die, N=112), a serotonin-norepinephrine reuptake inhibitor (SNRI), or placebo (N=125), for up to 8 weeks. Patients were excluded from the study if they suffered from bipolar disorder, schizophrenia and/or comorbid substance use disorder. Depressive symptoms were assessed using the Montgomery Åsberg Depression Rating Scale (MADRS) at baseline and at the end of the trial. The mean age was 46.8 years ± 12.8 SD.<br>The first replication cohort (Montréal cohort) was an independent group of 63 patients treated with citalopram, a selective serotonin reuptake inhibitor (SSRI), in an open-label trial. In this study, patients experiencing a MDE received citalopram for 8 weeks. The same exclusion criteria as above were applied.<br>Our second replication cohort (Marseille cohort) was a naturalistic prospective cohort that included 64 patients and 87 healthy controls. Patients were included during a MDE with HDRS-17 > 19 at the inclusion. Patients were excluded from this study if they suffered from bipolar disorder, schizophrenia and/or comorbid substance use disorder. All patients were treated at the inclusion with treatment as usual upon discretion of the treating psychiatrist. Healthy controls were free of any psychiatric disorder according to a semi-structured interview. All subjects included in the analysis were followed for 30 weeks with 4 points of evaluation (i.e. inclusion, 2 weeks later, 8 weeks later and 30 weeks later).<br>The post-mortem cohort was comprised of 75 post-mortem prefrontal cortex samples (Brodmann Area 44) . Brain pH and post-mortem interval (PMI) were used as tissue integrity measures and age and sex as co-variate. Subjects were either individuals who were suffering from a MDE at time of death by suicide (N=49), or psychiatrically normal controls (N=26), as assessed by psychological autopsies using DSM-IV criteria.
Recruitment	In the discovery cohort patients were recruited within clinical trials in different sites worldwide. Samples from these clinical trials were provided by Lundbeck by researchers blinded to our scientific hypothesis, the sole criterion being sample availability.<br>In the Montréal cohort all patients included in the study were recruited in Montréal (Douglas Mental health Institute). All samples were included in the analysis.<br>In the Marseilles cohort participants were recruited in different sites in France (Marseille, Montpellier, Tours, Clermont-Ferrand, Besancon, Nimes). Subjects were recruited in accordance to the inclusion/exclusion criteria described above, all included subjects were comprised in the analyses.<br>Post-mortem samples were obtained in collaboration with the Québec Coroner's office from the Douglas-Bell Canada Brain Bank (Douglas Mental Health University Institute, Montreal, Quebec, Canada).
Ethics oversight	This manuscript contains data from 4 registered clinical trials (www.ClinicalTrials.gov NCT00635219, NCT00599911, NCT01140906, NCT02209142). NCT00635219, NCT00599911, NCT01140906 were sponsored by Lundbeck and samples were provided as a donation to the Canadian Biomarker Integration Network in Depression (CAN-BIND) program. Additionally, the first replication cohort (Montréal) was conducted at the Douglas Mental Health Institute, Montréal, QC, Canada with the necessary approval from the hospital ethics and internal review board.<br>Ethics approval for the post-mortem study was obtained from the institutional review board of the Douglas Mental Health University institute.

Note that full information on the approval of the study protocol must also be provided in the manuscript.
